# Cocaine-induced locomotor sensitization associates with slow oscillatory firing of neurons in the ventral tegmental area

**DOI:** 10.1038/s41598-018-21592-7

**Published:** 2018-02-19

**Authors:** Chang-Liang Liu, Ya-Kun Wang, Guo-Zhang Jin, Wei-Xing Shi, Ming Gao

**Affiliations:** 1 Department of Pharmacology, Shanghai Institute of Materia Medica, Shanghai, 201203 China; 2 Department of Neurobiology, Barrow Neurological Institute, St. Joseph’s Hospital and Medical Center, Phoenix, AZ 85013 USA; 3 Departments of Pharmaceutical and Administrative Sciences, Loma Linda University Schools of Pharmacy and Medicine, Loma Linda, CA 92350 USA; 4 Cambrian Discovery Inc, Dover, MA 02030 USA

## Abstract

The initiation of psychostimulant sensitization depends on the mesocorticolimbic dopamine (DA) system. Although many cellular adaptations has been reported to be associated with this addictive behavior, the overall influence of these adaptations on the network regulation of DA neurons has not been established. Here, we profile a network-driven slow oscillation (SO) in the firing activity of ventral tegmental area (VTA) putative DA and non-DA neurons and their correlation with locomotor sensitization induced by repeated administration of cocaine. One day after the last cocaine injection, the power of SO (Pso) significantly increased both in DA and non-DA neurons. Interestingly, the Pso in DA neurons was positively correlated, while Pso in non-DA neurons was negatively correlated with the level of locomotor sensitization. On the other hand, the firing rates of DA and non-DA neurons were both elevated, but none exhibited any correlation with the level of sensitization. Fourteen days after the last injection, the Pso of DA neurons dissipated but still positively correlated with the level of sensitization. In contrast, the Pso in non-DA neurons lost correlation with locomotor sensitization. These results suggest that cocaine-induced locomotor sensitization is associated with long-term network adaptation in DA system and that DA and non-DA neurons may corporately facilitate/hamper the initiation of locomotor sensitization.

## Introduction

The mesocorticolimbic dopamine (DA) circuit, as the center for drug abuse and addiction^1,2^, undergoes long-term adaptations in response to repeated drug use, which promotes a risk of relapse after drug withdrawal^3^. Abusive drugs exert their rewarding effects, in part, by modulating the activity of DA and non-DA neurons within the ventral tegmental area (VTA)^4,5^. In rodents, repeated administration of many abusive drugs (i.e. cocaine, amphetamine and opioids) produces locomotor sensitization: a progressive and persistent augmentation of behavioral response to psychostimulants^6^. This behavioral phenomenon resembles some addiction-related features in humans^6,7^.

The development of locomotor sensitization is associated with increased activity in the mesocorticolimbic DA circuit, particularly in the VTA^8^. Several studies in brain slices have demonstrated that exposure to psychostimulants elicits drug-evoked synaptic plasticity in both excitatory and inhibitory inputs in VTA DA and non-DA neurons^3,9–12^. Another important mechanism that the altered activity is through intrinsic regulation of ion channels such as GABA_B_ receptor (GABA_B_R)-dependent G-protein-gated inwardly-rectifying K (GirK) channel^13,14^. Together, these neuroadaptations lead to significant alterations of firing activity in VTA neurons. VTA DA neurons fire in two patterns *in vivo*: irregular single-spike firing and burst firing^15–17^. Repeated cocaine administration results in an initial increase and later decrease over withdrawal time in the number of spontaneously active VTA DA neurons and in their mean firing rate^18,19^. It also facilitates the switch of DA neuron firing from single-spike to burst, which augments DA release in the VTA and nucleus accumbens^20,21^. These changes of firing activity in VTA DA neurons are believed to be involved in the development of behavioral sensitization induced by repeated cocaine exposure. Besides significant changes in DA neuronal activity, emerging evidence indicates that other brain regions also contribute to the initiation of behavioral sensitization, most likely through regulation of VTA neurons. For instance, ventral hippocampus activation increases DA neuronal population activity and facilitates locomotor sensitization^22^. By contrast, lesions of prelimbic cortex (PFC) prevent the development of behavioral sensitization to cocaine^23–25^.

An important gap in the research of neuronal plasticity in drug-induced behavioral sensitization is that most experiments are performed in brain slices, where important regulatory pathways are severed. These caveats make it difficult to evaluate the overall influence of the neural network on VTA neurons by repeated drug administration. Neuronal populations, via their anatomical and functional interconnections, can display sophisticated discharge patterns often in the form of oscillations that arise from the integration of network activity^26^. Using spectral analysis, we have reported an oscillatory activity at low frequency band (~0.8 Hz) in the firing sequences of VTA DA neurons under anesthesia, which is marked as slow oscillation (SO)^27^. The SO originates from the PFC and is under strong regulation of several brain areas, making it an ideal parameter to evaluate network regulation at a much bigger scale^28,29^. The SO in the firing of DA neurons is thought to increase DA release not only in more amounts, but also in specific temporal patterns^30^. Furthermore, oscillatory firing of DA neurons may also enable DA neurons to select inputs and regulate synaptic plasticity within the VTA^30^. VTA non-DA neurons also show SO in firing^29^, but its role in neural network and behavioral sensitization has been less well characterized. Here we measure the firing activity of DA and non-DA neurons in the VTA 1 day and 14 days after repeated injection of cocaine. We extract the SO from the firing sequences of these neurons and find a strong correlation between the power of SO and the level of behavioral sensitization.

## Methods

All experimental protocols were approved by and performed in accordance with guidelines set by the animal care and use and ethical committees at the Shanghai Institute of Materia Medica (Shanghai, China).

### Behavior measurement

**Figure 1:**
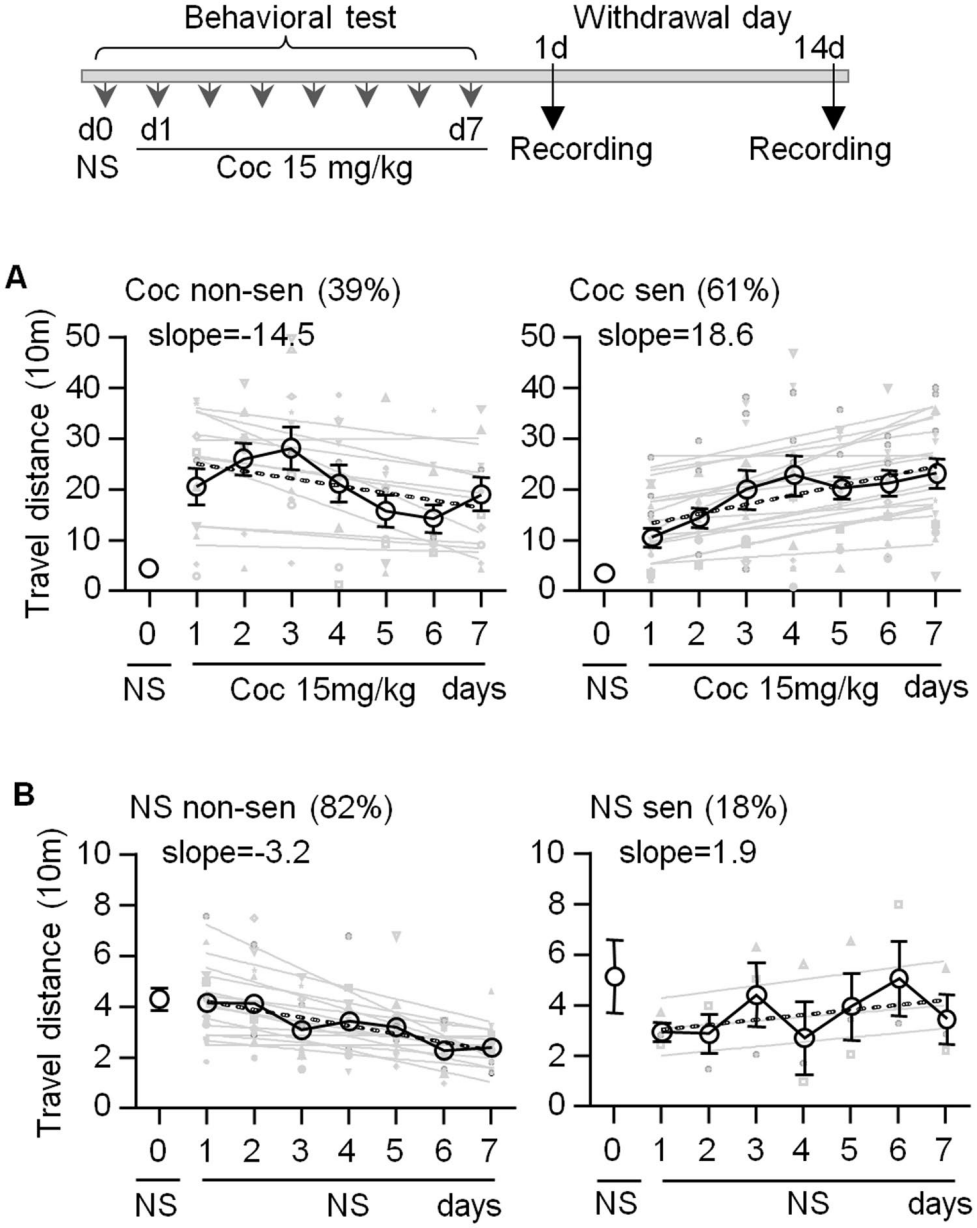
Repeated cocaine injections produce behavioral sensitization in a subset of rats. (**A**) Timeline and experimental design. (**B**) Locomotor activity data from all animals receiving saline injection (day 0) followed by repeated cocaine injection (day 1–7). These rats are classified as cocaine non-sensitized (left, n = 11 rats) and sensitized (right, n = 17 rats) group, according to the slope of linear regression of 7 days’ counts (slope: −14.5 vs 18.6). Each gray line indicates locomotor activity of one rat. The dashed trendline is calculated based on the black average line. (**B**) Locomotor activity data from saline group. Most rats are classified as non-sensitized group (left, n = 14, slope: −3.2).

Male Sprague Dawley rats, weighing 250–300 g at the start of the experiment, were housed in pairs with free access to food and water and were maintained in a 12-h light/dark cycle and temperature-controlled environment. Animals were allowed to acclimatize to the animal facility for at least 7 days following their arrival and were handled for 2–5 min daily. Locomotor activity was recorded with a video-camera-based activity monitoring system (Jiliang Soft Tech, China). All behavioral testing was conducted during the light cycle. Animal subjects were habituated to the activity chambers 2 h/day for two consecutive days prior to the experiments. On the day of experiment, all animals were habituated to the activity chamber for 40 min. After habituation, animals received either cocaine (15 mg/kg, i.p.; Qinghai Pharmaceutical Factory, China) or saline (1.0 ml/kg, i.p.), and subsequent behavior was monitored for 90 min. Behavioral tests were repeated for 8 days, including one injection of saline (day 0) and 7 injections of cocaine (day 1 to 7). Consistent with previous studies^31^, this cocaine treatment produced locomotor sensitivity in a subset of rats (Fig. 1). Single unit recording was conducted 1 day and 14 days after the last injection of cocaine.

### Single-unit recording

Rats were anesthetized with chloral hydrate (400 mg/kg, i.p., with supplemental doses administered via a lateral tail vein) and mounted in a stereotaxic apparatus. All pressure points and incision sites were infiltrated with the local anesthetic bupivacaine hydrochloride (0.25%; Harvest Pharmaceutical, Shanghai, China). Single-unit activities were recorded extracellularly using techniques that are similar to those described previously^29,32^. VTA putative DA neurons were identified by well established electrophysiological criteria^17,33–35^: biphasic (positive–negative) or triphasic (positive–negative–positive) spike waveform with a broad initial positive phase (>1.1 ms, measured from the start of action potential to the negative trough), an initial segment-somatodendritic break in the initial positive phase, and a slow firing rate (1–10 Hz, see suppl. Fig. 1). In addition, putative DA neurons can also be distinguished from non-DA neurons by the distinct low-pitch sound that are produced on an audio monitor. In all experiments, multiple cells were recorded from each animal using the “cells per track” technique^36^. 12 electrode tracks were made in the right side of the VTA [from lambda, anteroposterior (AP), 2.7–3.3; mediolateral (ML), 0.5–0.9; dorsoventral (DV), 7.0–8.5 mm]. Each track was separated by 200 μm. Throughout the experiment, body temperature was maintained at 36–38 °C with a homeothermic blanket system (Harvard Apparatus, Holliston, MA). At the end of the experiments, recording sites were marked by microiontophoresis of pontamine sky blue and were examined using standard histology methods. No difference in weight gain between cocaine- and saline-treated rats was observed in the treatment period.

### Data analysis

All data analyses were performed in Microsoft (Redmond, WA) Excel using in-house programs written in Visual Basic for Applications. The onset of a burst was identified as two consecutive spikes with an inter-spike interval (ISI) less than 80 ms, and the termination of a burst was defined as an ISI greater than 160 ms^16^. The variability of firing was estimated by the coefficient of variation (CV), which equals the SD of ISI divided by the mean (expressed as a percentage). Firing periodicity was examined using methods similar to those described previously^32^. Briefly, autocorrelograms (using autocovariance function with a bandwidth of 50 ms over 2048 bins) were constructed based on 2-min recordings selected from each cell. Following tapering using the Hanning-Tukey window function (15 windows with 50% overlap) and removing of the linear trend, the Fast Fourier Transform (FFT) was performed on autocorrelograms, yielding spectra with a resolution of 0.078 Hz. The amplitude of a spectral peak is expressed as a percentage of total power so that the sum of all peaks equals 100. Based on the relative amplitude of the slow oscillation (SO), VTA putative DA and non-DA neurons were divided into high- and low-SO (HS, LS) cells, respectively^27^. The HS cell was identified by the criterion: the mean spectral density between 0.5 and 1.5 Hz (P_0.5–1.5Hz_) was significantly higher than that between 0 and 3 Hz (P_0–3Hz_)^27^. The remaining VTA neurons were identified as LS cells because P_0.5–1.5Hz_ was not significantly higher than P_0–3Hz_.

Statistical significance of the comparison between two groups was assessed using two-tailed Student’s t-test. For statistical analysis of data from multiple groups, one-way ANOVA followed by *post hoc* Tukey tests was applied. Two-way ANOVA with time as the repeated measure was used to compare average locomotor activity counts of day 1–2 and day 6–7 of behavioral trials. Individual time points were compared between days using a post hoc Tukey’s test. Spectral data were log-transformed before being subjected to statistical comparison. The average of Pso, firing rate and bursting from recorded putative DA and non-DA neurons from each rat was represented as one point to run correlation with the level of behavioral sensitization. All values were based on analysis of 2 min recordings obtained under different conditions. All values were expressed as mean ± S.E.M, and P < 0.05 was considered significant.

## Results

### Behavioral sensitization in a subset of rats to repeated cocaine administration

Behavioral sensitization to repeated cocaine exposure in animals shows a progressive and enduring augmentation in locomotor^37^. To match with this definition and avoid single day variation of photocell counts on sensitization determination, locomotor activities were continuously collected for 1 hour after each injection of cocaine (15 mg/kg) for 7 days. Control rats received saline injections on all experimental days. Rats were considered to have developed behavioral sensitization if the slope of linear regression of 7 days’ locomotor activity was greater than zero. The treatment regimen used in the present study produced behavioral sensitization in 64% of the cocaine-treated animals, yet only 18% of saline-treated rats met the criterion for behavioral sensitization (Fig. 1A–C)^31^. On average, rats in sensitized groups (slope: 16.5 ± 2.4) displayed a linear increase in activity counts from day 1 to day 7. Rats in non-sensitized groups (slope: −14.5 ± 3.8) showed biphasic changes with an initial increase in counts during the first 3 injections (day 1–3) of cocaine and a gradual decrease after that (Fig. 1B). Further analysis showed that the average of photocell counts increased by over twofold on day 6–7 versus day 1–2 in sensitized rats, but decreased slightly in non-sensitized group (Suppl. Fig. 2). Interestingly, rats in non-sensitized groups showed more locomotor activity than those in sensitized groups to the injection of saline on day 0 (non-sensitized: 48.5 ± 4.2 m, n = 11, sensitized: 38.5 ± 1.8 m, n = 17, P < 0.05, t-test), suggesting a more sensitive response to external stimulation such as needle stimuli in non-sensitized groups.

### Enhanced oscillatory activity of VTA DA neurons in response to repeated cocaine exposure

**Figure 2:**
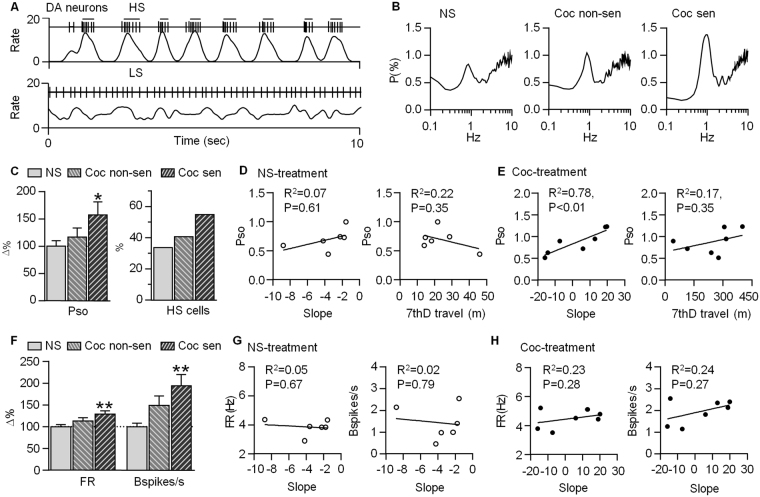
SO of VTA putative DA neuron firing significantly increased in sensitized rats 1 day after the last cocaine injection. (**A**) Two 10-s segments of spike trains (vertical bars) and corresponding smoothed, 50-ms-binwidth rate histogram (bottom line) of two typical DA cells: the up cell fires spikes in clusters in rhythmic manner at about 0.8 Hz, and is defined as high SO cell (HS). The bottom one fires in regular pattern and is defined as low SO cell (LS). (**B**) Average of power spectra are obtained from DA neurons recorded in saline-treated and cocaine-treated sensitized/non-sensitized rats. (**C**) Summary chart shows a significant increase of DA neuron Pso and percentage of HS cell. (**D**,**E**) Correlation of Pso to the slope of linear regression of 7 days’ counts and 7th day travel distance in saline-, and cocaine-treated rats, respectively. (**F**) Firing rate (FR) and bursting (Bspikes/s) increase in cocaine-treated sensitized rats. Neither has correlation with slope in saline-, and cocaine-treated groups (**G**,**H**). *P < 0.05, **P < 0.01 compared with saline group with One-Way ANOVA.

Slow oscillatory (SO) rhythms have been previously highlighted in spontaneous firing of VTA DA neurons, and have been characterized as network-mediated events^27^. To test whether the rhythmic firing pattern of VTA DA neurons was changed after repeated cocaine injection, firing activities of VTA putative DA and non-DA neurons were collected in each rat after the last injection, and oscillatory properties were determined by spectral analysis. In saline-treated rats (n = 6), 34% (32 of 95) of DA neurons showed a significant peak at around 0.8 Hz and were considered as high SO (HS) cells, which tended to fire repetitively in clusters (Fig. 2A–C). The low SO (LS) cells, by contrast, showed more regular firing (Fig. 2A). Repeated cocaine treatment significantly increased the power of SO (Pso) and percentage of HS cells in cocaine sensitized rats (Pso: F_2,165_ = 3.9, P < 0.05, One-Way ANOVA; HS% in non-sensitized (n = 3 rats) and sensitized group (n = 4 rats): 41% vs 55%, Fig. 2C, Table 1). This experiment suggests that repeated cocaine treatment potentiates the oscillatory firing of VTA DA neurons.

**Table 1:** Firing activities of VTA putative DA neuron.

Item		FR	Bspikes/s	Pso	ISI CV	HS (%)	Cell^#^	Rat^#^
naive rat		3.78 ± 0.11	1.18 ± 0.09	0.97 ± 0.08	75.75 ± 1.65	45%	245	17
NS 1d		3.74 ± 0.16	1.15 ± 0.14	0.65 ± 0.07^&^	78.41 ± 2.96	34%	95	6
Coc 1d	non-sen rat	4.24 ± 0.29	1.71 ± 0.25	0.70 ± 0.09	77.49 ± 2.98	41%	31	3
	sen rat	4.82 ± 0.27**	2.23 ± 0.30***	1.02 ± 0.16*	75.06 ± 2.89	55%	42	4
NS 14d		3.44 ± 0.18	1.24 ± 0.14	0.96 ± 0.08	82.37 ± 2.43	46%	79	5
Coc 14d	non-sen rat	3.87 ± 0.22	1.16 ± 0.23	0.69 ± 0.10	73.55 ± 3.55	47%	37	4
	sen rat	3.55 ± 0.24	1.07 ± 0.20	0.79 ± 0.13	78.40 ± 4.51	44%	43	5

^*^P < 0.05, ^**^P < 0.01, ^*^^**^P < 0.001 compared with saline-treated group (NS 1d) on day 1 withdrawal time by One-way ANOVA followed by *post hoc* Tukey tests. Multiple groups: NS1d vs Coc 1d non-sen rat vs Coc 1d sen rat. Because less DA cells were collected, one rat from saline-treated group and another one from cocaine group were not involved when running correlations (see Fig. 2E–H and Fig. 3D–G).

^&^P < 0.05 compared with naive group by One-way ANOVA followed by *post hoc* Tukey tests. Multiple groups: Naive vs NS1d vs NS14d.

To better investigate the relationship between SO and the level of cocaine-induced locomotor sensitization, we plotted the Pso against the level of behavioral sensitization for each rat. The Pso was calculated by averaging the power spectra of all cells from a particular animal and the level of sensitization was quantified by the slope of the linear regression of 7 days’ photocell counts. Pso showed a strong positive correlation with the level of locomotor sensitization in cocaine-treated rats, but had no correlation in saline-treated rats (saline group: R^2^ = 0.07, P = 0.61; cocaine group: R^2^ = 0.78, P < 0.01, Fig. 2D,E). To rule out the possibility that Pso was simply correlated with the effect of cocaine on locomotor activity, we plotted the Pso against the locomotor activity on the last injection of cocaine, but found no correlation between the two (saline group: P = 0.35; cocaine group: P = 0.35, Fig. 2D,E). Given that the SO is purely a product of network input^26^, this experiment suggests that enhancement of network regulation may underlie the development of cocaine-induced behavioral sensitization.

Repeated cocaine administration has been reported to induce a significant increase in the firing activity of VTA individual DA neurons^18^. Consistently, 1 day after the last cocaine injection, both firing rate (FR) and bursting (the number of spikes occurring in bursts, Bspikes/s) of VTA DA neurons were significantly increased in cocaine-treated compared to saline-treated rats (FR: F_2,165_ = 6.7, P < 0.01; Bspikes/s: F_2,165_ = 9.9, P < 0.001, Fig. 2F). However, neither firing rate nor bursting of VTA DA neurons correlated with the level of locomotor sensitization in saline-treated groups (FR: P = 0.67; Bspikes/s: P = 0.79; Fig. 2G) or cocaine-treated rats (FR: P = 0.43; Bspikes/s: P = 0.27, Fig. 2F). This suggests that the traditional measures, which represented the firing activity of individual DA neurons, were not enough to reflect the behavioral sensitization.

### Enhanced oscillatory activity of VTA non-DA neurons in response to repeated cocaine exposure

**Figure 3:**
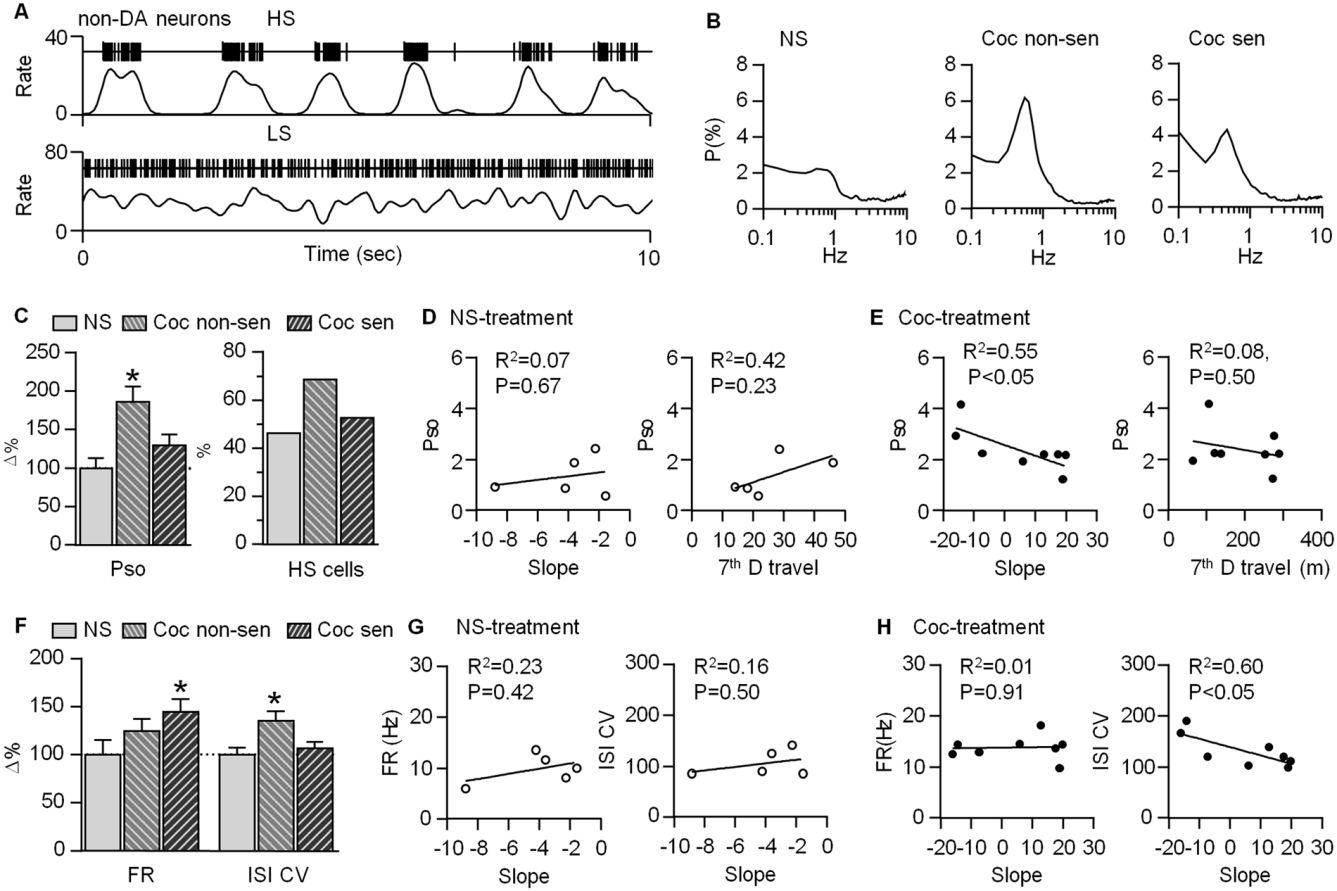
SO of VTA non-DA neuron firing significantly increased in non-sensitized rats 1 day after the last cocaine injection. (**A**) Two 10-s segments of spike trains (vertical bars) and corresponding smoothed, 50-ms-binwidth rate histogram (bottom line) of two typical non-DA cells. Similar to DA neuron, non-DA neuron also presents cluster firing in a SO manner at about 0.6 Hz. The up one shows high oscillatory firing, but bottom one shows low SO. (**B**) Average of power spectra are obtained from non-DA neurons recorded in the same batch of saline-treated (NS) and cocaine-treated sensitized/non-sensitized rats on day 1 withdrawal time. (**C**) Summary chart shows a significant increase of non-DA neuron Pso, and high percentage of HS cell in non-sensitized group. (**D**,**E**) Correlation of Pso to the slope of linear regression of 7 days’ counts and 7th day travel distance in saline-, and cocaine-treated rats, respectively. (**F**) Firing rate and ISI CV increase in sensitized and non-sensitized group, respectively. (**G**) Neither firing rate nor ISI CV has correlation with the slope in saline-treated group. H) ISI CV, not firing rate, has a significant negative correlation with slope in cocaine-treated group. *P < 0.05, **P < 0.01 compared with saline group with One-Way ANOVA.

The VTA non-DA neurons (mainly GABAergic neuron) are thought to tonically inhibit local DA neurons within the VTA to keep this microenvironment in a balance. In this present study, recorded neurons which did not meet the criterion for putative DA neurons were classified as non-DA neurons. Our previous studies show that many VTA non-DA neurons fire in a rhythmic manner under anesthesia (Fig. 3A) and synchronize with negative deflections of VTA DA neurons^29^. Day 1 after the last cocaine injection, Pso of non-DA neurons significantly increased in non-sensitized rats, but had no change in sensitized rats compared with the saline-treated group (F_2,144_ = 7.1, P < 0.01, Fig. 3B,C, Table 2). 46% of non-DA neurons in the saline-treated group fired with high SO (Fig. 3C). Interestingly, in the cocaine-treated group, the percentage of the HS cell in non-sensitized rats was much higher than that observed in sensitized rats (68% vs 53%, Fig. 3C). Pso of non-DA neurons showed no correlation with the level of locomotor sensitization in saline-treated groups, but had a significant negative correlation in cocaine-treated rats 1 day after the last injection (saline group: P = 0.67; cocaine group: P < 0.05, Fig. 3D,E). Further analysis showed that Pso had no correlation with locomotor activity on the 7th-day cocaine injection (saline group: P = 0.23; cocaine group: P = 0.50, Fig. 3D,E). These results suggest that the level of rhythmic firing of VTA non-DA neurons might counteract the development of behavioral sensitization induced by cocaine.

**Table 2:** Firing activities of VTA non-DA neuron.

Item		FR	Pso	ISI CV	HS (%)	Cell^#^	Rat^#^
naive rat		10.10 ± 0.64	1.87 ± 0.12	128.87 ± 4.82	44%	190	17
NS 1d		10.62 ± 1.62	1.60 ± 0.21	114.36 ± 8.52	46%	50	5
Coc 1d	non-sen rat	13.22 ± 1.38	2.97 ± 0.32^***^	154.54 ± 12.13^*^	68%	38	3
	sen rat	15.43 ± 1.38^*^	2.07 ± 0.22	121.83 ± 8.44	53%	59	5
NS 14d		9.25 ± 0.81	1.85 ± 0.21	120.71 ± 6.30	45%	51	5
Coc 14d	non-sen rat	10.18 ± 1.24	1.39 ± 0.21	100.86 ± 6.03	30%	43	4
	sen rat	10.55 ± 1.20	2.61 ± 0.23^#^	138.17 ± 7.27	70%	50	5

^*^P < 0.05, ^***^P < 0.001 compared with saline-treated group (NS 1d) on day 1 withdrawal time by One-way ANOVA followed by *post hoc* Tukey tests. Multiple groups: NS1d vs Coc 1d non-sen rat vs Coc 1d sen rat.

^#^P < 0.05 compared with saline-treated group (NS 14d) on day 14 withdrawal time by One-way ANOVA followed by *post hoc* Tukey tests. Multiple groups: NS14d vs Coc 14d nonsen rat vs Coc14d sen rat.

In 190 non-DA neurons recorded from naive rats (n = 17), firing rate varied within a huge range from 1 Hz to 50 Hz. On average, the frequency was 10.1 ± 0.6 Hz. To test whether the non-DA neurons changed their firing activity after repeated cocaine exposure, their properties were compared between sensitized and non-sensitized rats. Basal firing rate of non-DA neurons increased in cocaine-treated rats, but only significantly changed in sensitized rats as compared to saline treated group (F_2,144_ = 2.9, P < 0.05). However, the inter-spike interval coefficient of variation (ISI CV) significantly increased in non-sensitized rats (F_2,144_ = 4.4, P < 0.05, Fig. 3F). Similar to DA neurons, neither firing rate nor ISI CV of VTA non-DA neurons correlated with the level of locomotor sensitization in saline-treated groups 1 day after the last injection (FR: P = 0.42; ISI CV: P = 0.50, Fig. 3G). On the other hand, ISI and CV exhibited a significant negative correlation with locomotor sensitization in cocaine-treated groups (ISI CV: P < 0.05; FR: P = 0.91, Fig. 3H) consistent with the reduced Pso of VTA non-DA neurons in cocaine-induced behavioral sensitization.

### Oscillatory properties of VTA DA and non-DA neurons 14 days after the last cocaine injection

**Figure 4:**
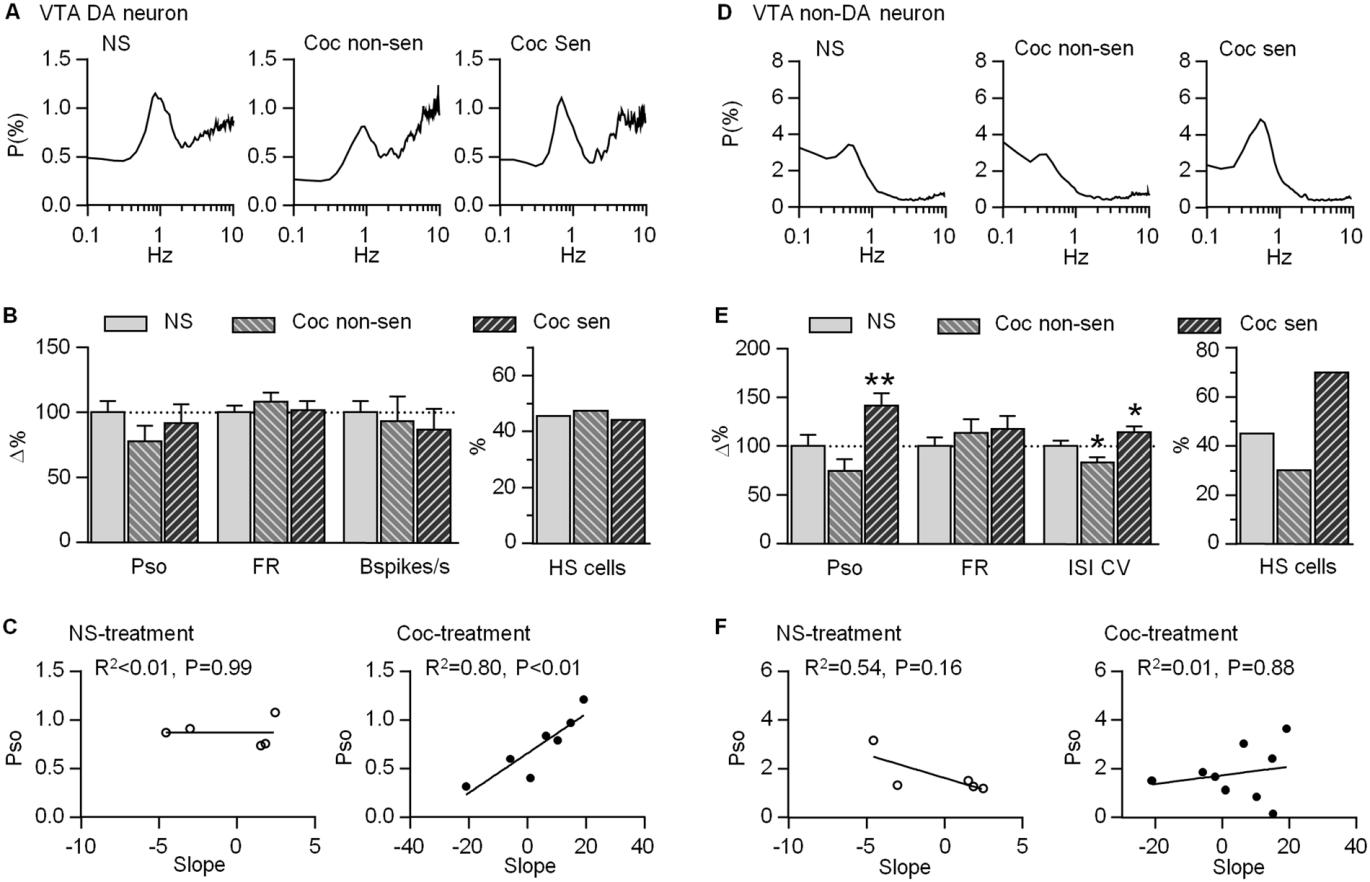
Profiles of firing activity of VTA putative DA and non-DA neurons 14 days after the last cocaine injection. (**A**) Average of power spectra are obtained from DA neurons recorded in saline-treated (NS) and cocaine-treated sensitized/non-sensitized rats on day 14 withdrawal time. (**B**) Summary chart shows no significant change of DA neuron firing activities and similar percentage of HS cell among three groups. (**C**) Pso shows a significant positive correlation with the slope in cocaine-, not saline treated group. (**D**) Average of power spectra are obtained from non-DA neurons recorded in the same batch of saline-treated (NS) and cocaine-treated sensitized/non-sensitized rats on day 14 withdrawal time. (**E**) Summary chart shows a significant increase of non-DA neuron Pso and ISI CV in cocaine-treated sensitized rats (left). Less percentage of HS cell is found in non-sensitized rats, but more in sensitized group (right). (**F**) Pso shows no correlation with slope in both saline- and cocaine-treated group. *P < 0.05 compared with saline group with One-Way ANOVA.

Psychostimulants-induced behavioral sensitization and associated neuroadaptations persist over a long period of time after the withdrawal. To determine whether oscillatory properties of VTA neurons dissipated over time, the firing of VTA neurons were collected 14 days after the last cocaine injection. The percentage of HS cell in VTA DA neurons of the three groups were almost the same, and Pso of DA neurons showed no significant difference (F_2,157_ = 0.92, P = 0.40, Fig. 4A,B). Interestingly, the Pso of cocaine-treated group still showed significant correlation with the level of locomotor sensitization, but the correlation was not observed in the saline group (cocaine group: P < 0.01; saline group: P = 0.99, Fig. 4C). Firing rates and bursting changed comparatively as compared to saline-treated rats (FR: F_2,157_ = 0.44, P = 0.64; Bspikes/s: F_2,157_ = 0.29, P = 0.75, Fig. 4B), and showed no correlation with locomotor sensitization in saline-treated group (FR: P = 0.67; Bspikes/s: P = 0.64; Suppl. Fig. 3) and cocaine-treated group (FR: P = 0.07; Bspikes/s: P = 0.87, Suppl. Fig. 5).

In the non-DA neurons that were recorded 14 days after the last injection, their firing rates had no significant difference (FR: F_2,141_ = 0.77, P = 0.47), but their firing patterns significantly changed. The percentage of the HS cell was much lower in non-sensitized rats (HS: 30%), but much higher in sensitized rats (HS: 70%) compared to that of the saline-treated group (HS: 45%). Pso of non-DA neurons decreased in non-sensitized rats, but significantly increased in sensitized rats (F_2,141_ = 11.78, P < 0.001). Similarly, ISI CV significantly decreased in non-sensitized rats, but increased in sensitized rats compared to that of saline-treated group (Pso: F_2,141_ = 12.60, P < 0.001, Fig. 4E). Further analysis showed that Pso and firing rate had no correlation to locomotor sensitization in saline- and cocaine-treated rats (saline group: Pso: P = 0.16; FR: P = 0.17; cocaine group: Pso: P = 0.88, FR: P = 0.81, Fig. 4F, Suppl. Fig. 5).

### Correlation of firing activity between DA and non-DA neurons within the VTA

**Figure 5:**
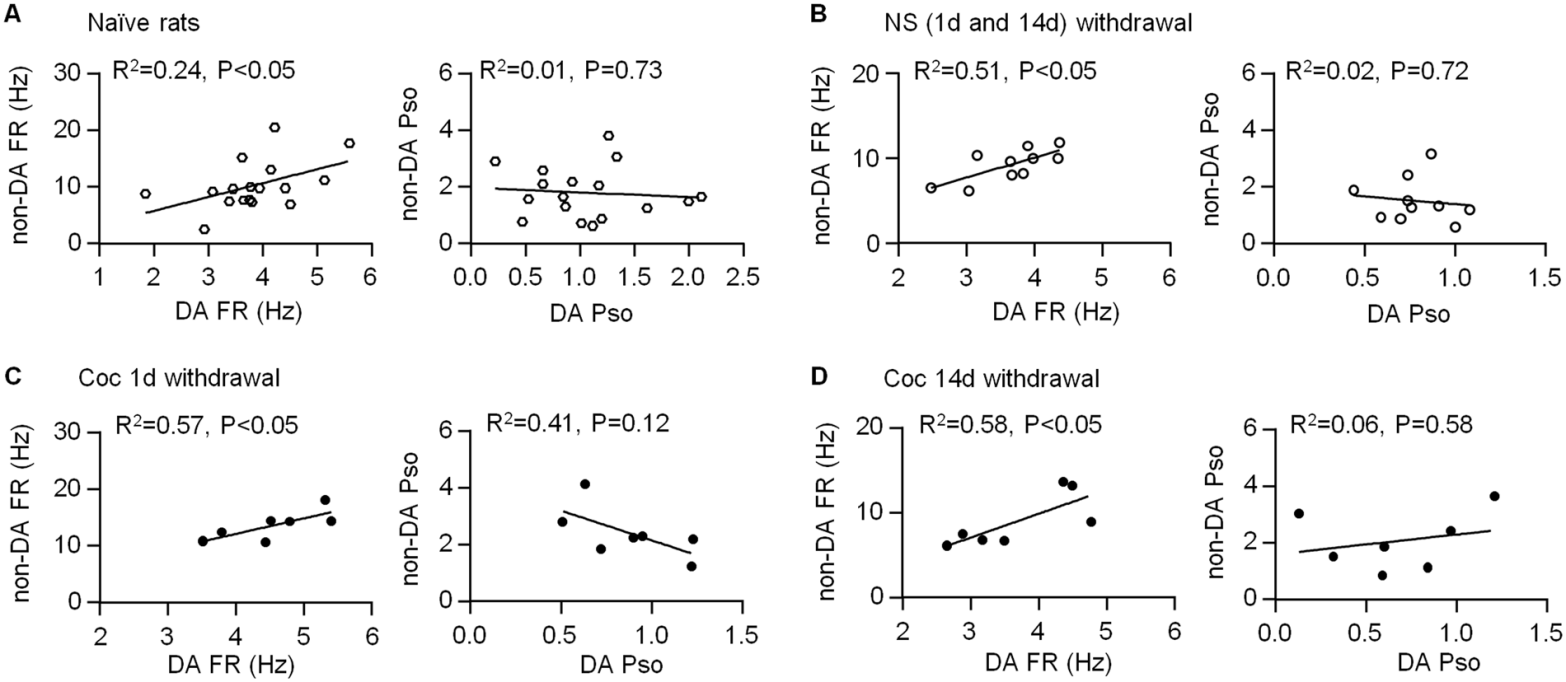
Relationship of firing measures between VTA putative DA and non-DA neurons on days 1 and 14 withdrawal time. (**A**) Firing rate (FR), not Pso, shows a significant positive correlation between DA and non-DA neurons in naive rats. (**B**) Firing rate, not Pso, still has a positive correlation between DA and non-DA neurons in saline-treated, or cocaine-treated rats 1 day and 14 days after the last injection. The data in saline-treated group on day 1 and 14 withdrawal time are pooled together.

GABA neurons tonically inhibit firing activities of the surrounding DA neurons within the VTA. The present study showed that both putative DA and non-DA neurons significantly increased their firing rates 1 day after the last cocaine injection, suggesting a potential interaction between them during behavioral sensitization. We thus examined the correlation of firing activities between VTA DA and non-DA neurons in each animal. In the naive rats, average firing rate of DA neurons showed a significant positive correlation with that of non-DA neurons (FR: P < 0.05, n = 17 rats, Fig. 5A). Pso, by contrast, had no correlation (Pso: P = 0.73). To test whether repeated cocaine administration would affect these correlations, we compared data collected from 1 and 14 days after last cocaine injection. To increase the sample size of saline-treated group, we pooled data collected on Day 1 and Day 14 after the last saline injection. The results showed that firing rate, but not Pso, still had a significant correlation between putative DA and non-DA neurons (FR: P < 0.05; Pso: P = 0.72, n = 12 rats, Fig. 5B). Similarly, in rats treated with cocaine, there was a significant positive correlation in firing rate between DA and non-DA neurons (Fig. 5C,D). There was a trend of negative correlation in the Pso 1 day but not 14 days after last cocaine administration, but it did not reach significance (Fig. 5C). These results suggest that firing rates between putative DA and non-DA neurons are positively correlated independent of cocaine exposure.

## Discussion

Drug-evoked firing synchronization of VTA neurons causes persistent modifications in the neural circuits and eventually leads to behavioral sensitization. We report here that repeated cocaine injections selectively enhance Pso in the firing sequences of VTA DA and non-DA neurons after 1 day of withdrawal, which may contribute to sensitized and non-sensitized conditions. Furthermore, repeated cocaine exposure leads to changes in the traditional firing measures of these two cell populations from an initial significant increase to a later recover to baseline over withdrawal. Interestingly, the firing rate of DA and non-DA neurons are always positively correlated with each other at different stages, indicating that the elevated firing activity of DA neurons after cocaine administration is not caused by local disinhibition. Instead, it may represent an attempt to maintain a balance at a new set point in excitatory-inhibitory circuit within the VTA.

Psychostimulant-induced excitation in VTA DA neurons has been studied extensively. In lateral VTA, DA and GABA neurons make up the majority of neurons (~65% vs ~30%)^38^. VTA DA neurons can be reliably identified *in vivo* by their specific characteristics such as slow waveform and large trough segment (≥1.1 ms)^18,33,35,39^. They exhibit two firing patterns *in vivo*- tonic and phasic^15,16^, which contribute to a background low DA level and an information-encoding high DA level in downstream regions, respectively^40,41^. A host of cellular neuroadaptations, including synaptic and intrinsic changes, elicited by repeated exposure of cocaine likely contribute to the elevated activity of VTA DA neurons after repeated drug expsorue^10,11,13,14^. After 7 days’ integration of synaptic and intrinsic regulation *in vivo*, repeated cocaine injections significantly enhance the traditional firing activities in VTA DA neurons on the first day withdrawal, and tends to fall back to baseline or even lower after 14 days of withdrawal. The level of firing enhancement in sensitized rats is higher than that of non-sensitized rats. Our findings are consistent with previous reports^18,19^. Further analysis shows that firing rate and bursting have no correlation with the level of behavioral sensitization in saline- and cocaine-treated rats. This suggests that the increase of firing rate and bursting may facilitate, but not be sufficient for, the development of cocaine-induced behavioral sensitization.

Non-DA neurons, in the present study, are mostly GABAergic inhibitory neurons^38^, as glutamatergic neurons largely localize in the medial VTA^42^. Many addictive drugs inhibit VTA GABA neurons, which disinhibit DA neuron activity in the acute phase^12,43–46^. However, after repetitive exposure, addictive drugs may increase VTA GABA neuron firing by depressing GABA_B_R signaling, thus enhancing GABA mediated inhibition of DA neurons^14,45,47^. In the present study, the increase in DA firing rate and bursting does not seem to result from disinhibition from local GABAergic input, because firing rate of non-DA neurons is always positively correlated with that of DA neurons in naive, saline-, and cocaine-treated rats. Rather, the strong positive correlation in the firing may represent an attempt to counteract the excitation of DA neurons.

Slow oscillations reflect widespread ‘up’ and ‘down’ states of synchronized network activity, engaging neurons throughout the cortex and subcortical areas^48^. The SO activity reliably generates in anesthetized experimental preparations, but its frequency varies depending on different anesthetics^49^. For example, ketamine-xylazine tends to accelerate the rhythm (0.6–1.0 Hz) by reduction of hyperpolarization, however, urethane produces a slower rhythm (0.3–0.4 Hz) because of multiple effects on neurotransmitter-gated ion channels^50,51^. In the VTA, half of DA and non-DA neurons present SO in firing activity in naive animals under chloral hydrate anesthesia condition^29^. The SO of VTA neurons is generated within the PFC, and can be strongly regulated by psychostimulants^27,29^. In the present study, the percentage of HS cell and Pso are clearly higher specifically in the VTA putative DA neurons in cocaine sensitized rats 1 day after the last injection of cocaine, indicating an elevated network regulation. Furthermore, Pso is positively correlated with the level of locomotor sensitization in cocaine-treated rats. This suggests that enhanced oscillatory firing of the VTA putative DA neurons may facilitate the development of cocaine-induced behavioral sensitization. Our finding is supported by the facts that PFC lesion or inactivation not only inhibits the SO of putative DA neuron firing, but also prevents the development of sensitization induced by repeated systemic administration of cocaine^23–25^. Our results thus indicate that repeated injections of cocaine may strengthen input drive from widely distributed brain areas including the PFC and promote synchrony between functionally related neurons within circuits.

Previous studies indicate that VTA GABA neurons also receive inputs from the PFC and subcortical areas that could provide reward-related signals^46,52,53^. Phasic excitation of VTA GABA neurons could be driven by inputs from lateral habenula neurons that are phasically excited by aversive stimuli^46^. Our study shows that the strong oscillatory firing in the VTA non-DA neurons is negatively correlates with locomotor sensitization following repeated injections of cocaine. This suggests that VTA non-DA neurons provide an inhibition to counteract excitatory drive from primary reward caused by repeated injections of cocaine. The non-sensitized rats exhibit biphasic response to cocaine injection, with an initial rapid excitation followed by delayed inhibition on travel distance; the sensitized groups exhibit linear increase in response. Furthermore, these rats are susceptible to external stimuli showing more locomotor activity to day 0 saline injection. Thus, the initial treatment of cocaine may produce a maximum effect on oscillatory firing of VTA DA neurons, further injection results in an inhibition by dominating strong oscillation of non-DA neurons. The biphasic response may be similar to cocaine’s shift from rewarding to aversive effect in a dose-dependent manner^54^, and cause these animals in a non-sensitized state. This finding may help explain the phenomena of bell-shaped dose-response curves for almost all self-administered drugs^54^. After 14 days’ withdrawal, Pso is getting lower in VTA putative DA neurons, but higher in non-DA neurons in sensitized rats than that in saline-treated group, which may cooperate together to produce aversive effects after initial rewarding effects dissipate^55,56^. However, the exact role of VTA non-DA neurons in cocaine-induced behavioral sensitization needs further investigation.

Many studies have demonstrated effects of environmental stimuli on locomotor activity produced by daily injection. Such an effect by saline has been noted previously in which saline pretreatment is shown to alter the behavioral response to subsequent exposure to stress^57^. These authors postulate that a saline injection may function as a mild stressor, and based upon suggestions that the behavioral and neurochemical effects of stress and psychostimulants may be interchangeable^58^. Present results show that the traditional firing measures have not changed, but Pso decreases in DA and non-DA neurons on day 1 withdrawal following repeated injection of saline, suggesting that SO is likely a more sensitive parameter, and that daily injection did produce aversive stimuli on neural circuits of VTA neurons within a short withdrawal time.

In summary, these data demonstrate that behavioral sensitization to cocaine is associated with long-term changes in oscillatory firing of VTA neurons. The strength of SO of VTA putative DA and non-DA neurons correlates with the initiation of behavioral sensitization in a positive and negative manner, depending on the sensitivity of individual animal to cocaine administration. Our study helps understand how DA and non-DA neurons within the VTA simultaneously orchestrate behavioral responses to cocaine administration, and also highlight the significance of SO of VTA neurons in psychostimulant-induced behavioral sensitization. Furthermore, this provides a circuit mechanism through which suppression of SO in the VTA might reduce the rewarding effects of cocaine.

## Electronic supplementary material

Supplemetary Information
