# Supplementary material for: Cocaine-induced locomotor sensitization associates with slow oscillatory firing of neurons in the ventral tegmental area
Source: Sci Rep. 2018 Feb 19;8:3274. doi: 10.1038/s41598-018-21592-7 (PMC5818474; doi:10.1038/s41598-018-21592-7)

Cocaine-induced locomotor sensitization associates with slow oscillatory firing of neurons in the ventral tegmental area

Chang-Liang Liu[1,4], Ya-Kun Wang[2], Guo-Zhang Jin[1], Wei-Xing Shi[1,3], Ming Gao[1,2]*

Supplementary Figure

Supplementary Fig. 1. Characteristics of action potential and vertical depth of recorded VTA putative DA and non-DA neuron from naive rats. A) Representative action potential (AP) waveform of putative DA neuron (left) and non-DA neuron (right). The dashed lines indicate the definition of AP width and duration in our study. B) A 2D plot of AP width vs firing rate from putative DA and AP duration vs firing rate from non-DA neurons. The averages of AP width of putative DA is 1.67 ± 0.03 ms (n = 103) and AP duration of non-DA neurons is 1.25 ± 0.03 ms (n = 154). C) One recording track in VTA. D) Distribution of vertical depth of recorded putative DA and non-DA neurons.

Supplementary Fig. 2. Comparison of average of locomotor activity between on day 6-7 and on day 1-2. Sensitized rats showed more locomotor activity on average of day 6-7 (d6-7) versus day 1-2 (d1-2, B), however, non-sensitized rats showed less (A). **P < 0.01; *P < 0.05 for comparison between photocell counts on average of day 1-2 versus day 6-7 with Two-way ANOVA.

Supplementary Fig. 3. Distributions of firing rate and bursting of VTA putative DA and non-DA neurons between saline and cocaine treatment from 1d (A) and 14d withdrawal (B).

Supplementary Fig. 4. Relationship between firing rate and bursting, firing rate and Pso, bursting and Pso of VTA putative DA neurons from naive rats (A), saline 1d and 14d withdrawal (B), cocaine 1d withdrawal group (C). The results suggested that firing rate of putative DA neurons always showed a high correlation with bursting. The average of Pso, firing rate and bursting from recorded putative DA and from each rat was represented as one point to run correlation.

Supplementary Fig. 5. Relationship between slope of linear regression of 7 days' counts to firing measures of VTA neurons 14 day after the last injection. A) Firing measures (FR and Bspikes/s) versus slope from putative DA neurons in saline- and cocaine-treated rats, showing no significant correlation on day 14 withdrawal time. B) FR versus slope from non-DA neurons in saline-, or cocaine-treated rats, showing no correlation.

Supplementary Figure-1

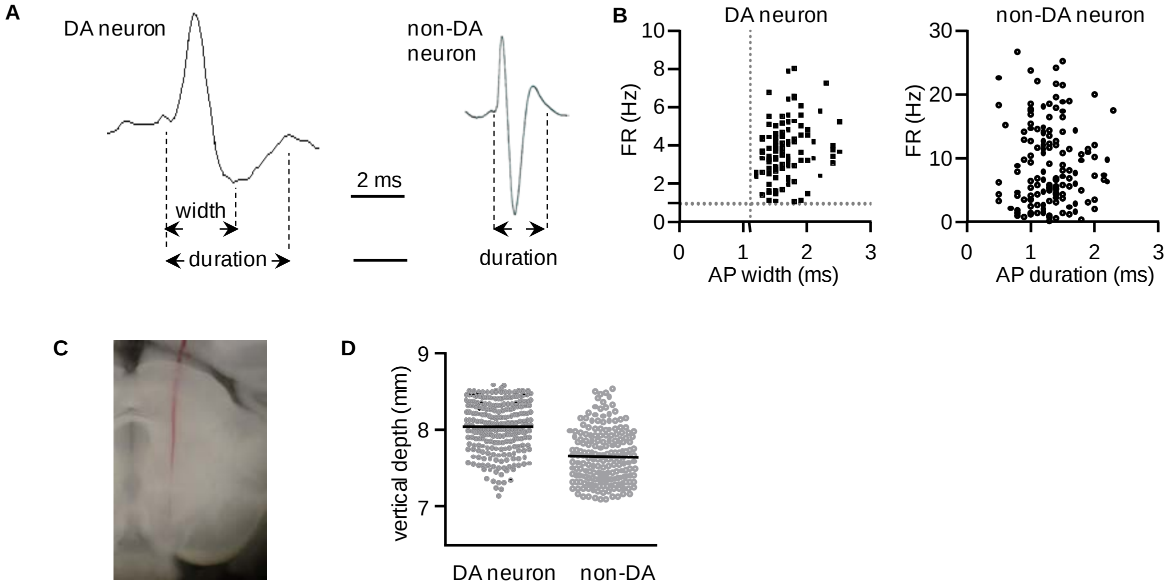

Supplementary Figure-2

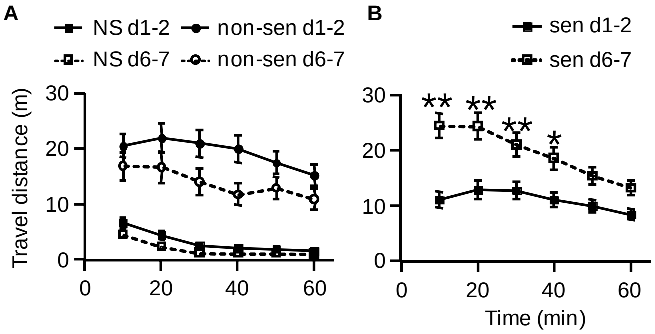

Supplementary Figure-3

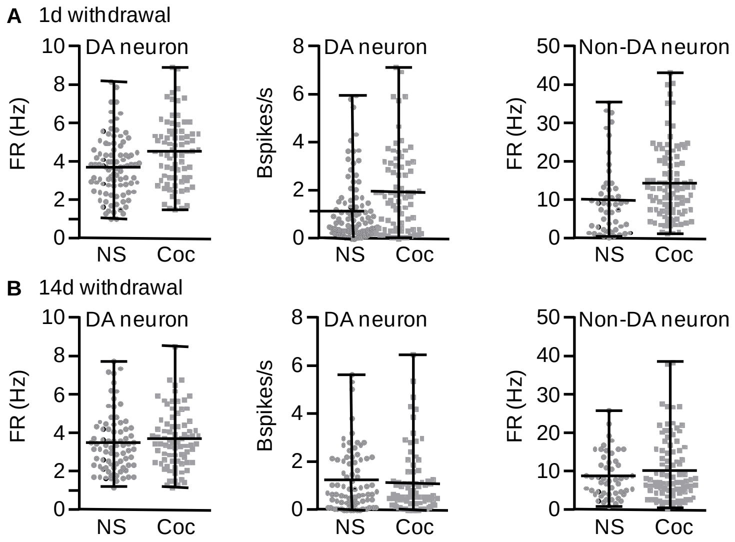

Supplementary Figure-4

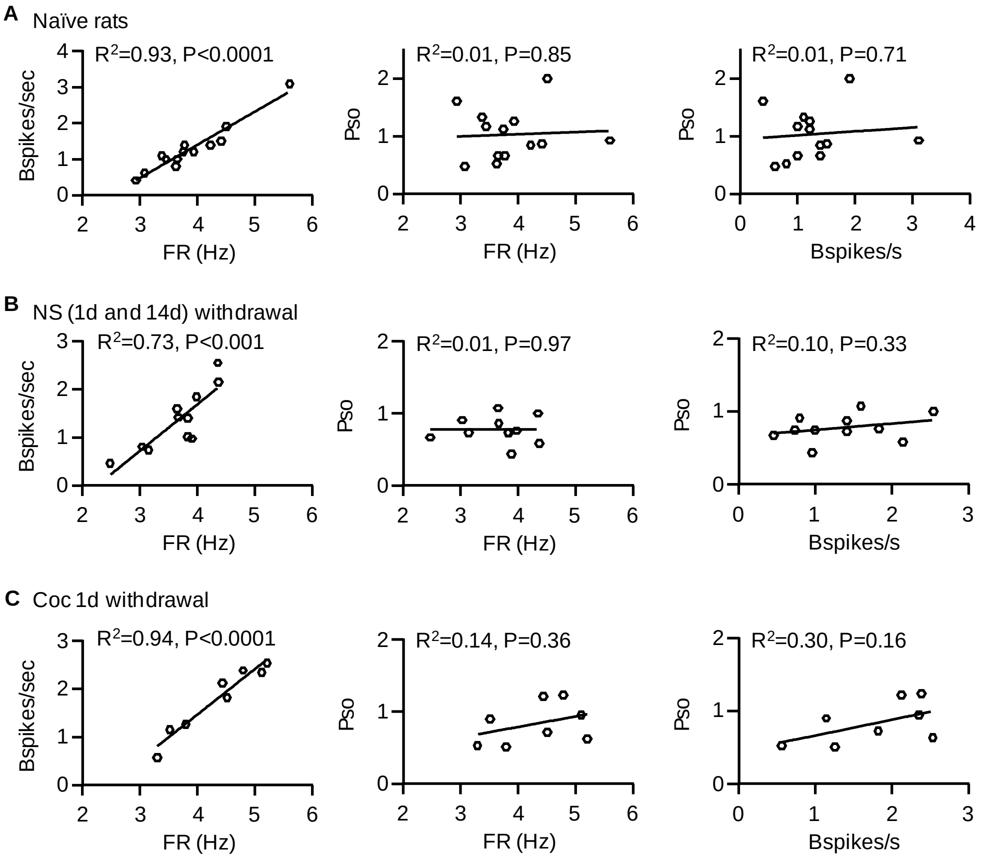

Supplementary Figure-5

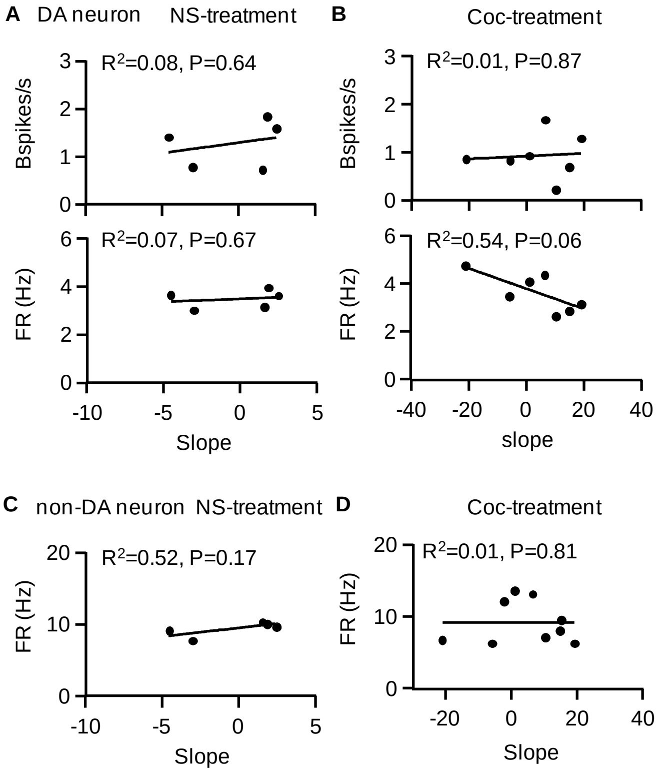

Supplement: Supplementary file 1 — Supplemetary Information [file 41598_2018_21592_MOESM1_ESM.pdf]
